# Failure Mechanisms of the Coating/Metal Interface in Waterborne Coatings: The Effect of Bonding

**DOI:** 10.3390/ma10040397

**Published:** 2017-04-09

**Authors:** Hongxia Wan, Dongdong Song, Xiaogang Li, Dawei Zhang, Jin Gao, Cuiwei Du

**Affiliations:** 1 Institute for advanced Materials and Technology, University of Science and Technology Beijing, Beijing 100083, China; wanhongxia88@163.com (H.W.); dawzhang@126.com (D.Z.); g.jin@163.com (J.G.); dcw@ustb.edu.cn (C.D.); 2 Aerospace Research Institute of Materials and Processing Technology, Beijing 100076, China; 3 Ningbo Institute of Material Technology and Engineering, Chinese Academy of Sciences, Ningbo 315201, China

**Keywords:** waterborne coating, scanning electrochemical microscopy (SECM), delamination

## Abstract

Waterborne coating is the most popular type of coating, and improving its performance is a key point of research. Cathodic delamination is one of the major modes of failure for organic coatings. It refers to the weakening or loss of adhesion between the coating and substrate. Physical and chemical characteristics of coatings have been studied via scanning electron microscopy (SEM), atomic force microscopy (AFM), contact angle measurements, Fourier transform infrared spectroscopy (FTIR), and secondary ion mass spectrometry (SIMS). Early heterogeneous swelling at the metal-coating interface in non-defective coated metals was elucidated using frequency-dependent alternating-current scanning electrochemical microscopy. Two types of coatings (styrene-acrylic coating and terpolymer coating) were compared. The effects of thickness, surface roughness, and chemical bonding on cathodic delamination were investigated.

## 1. Introduction

Organic coating is the most effective and economical method to protect metallic materials from corrosion [1,2,3,4,5,6]. The protective coating can isolate the metal substrate from corrosive media [7], provide electrochemical protection (inhibition [8] and cathodic protection [9]), and exhibit an adhesive function [10]. However, organic coatings degrade under aggressive environments because of underfilm corrosion, which can shorten the service life of coatings [11,12,13,14,15]. Degradation is accelerated by scratches or wear, allowing the diffusion of aggressive media to the interface between the coating and metal. Studies [10,16] have shown that the main reason for the anti-corrosion role of coatings is not physical isolation, but the adhesion of coatings is fundamental to its protective effects.

The process and mechanism of coating swelling has been investigated by numerous studies. Stratmann [17,18,19] studied, in situ, the delamination characterization on coating defects and obtained the kinetics and the electrochemical model of the delamination using the Scanning Kelvinprobe (SKP) method, a mechanical test with a homemade device and infrared analysis. McMurray et al. [20,21,22] used the SKP method to study the effect of an inhibitor on corrosion in the coating/metal interface; results showed that the inhibitor in the coating can slow down oxygen reduction and underfilm corrosion, thereby relieving delamination in the coating. Santana et al. [23] studied the early specific effect of chloride ions on heterogeneous swelling at the metal-polymer interface using frequency-dependent alternating current-scanning electrochemical microscopy (AC-SECM). Souto reported changes in coatings induced by chloride ions in situ during immersion [24,25,26]. The majority of research focused on the relationship between aggressive environment and coating failure. However, studies on the effect of the adhesion mechanism on coating swelling are extremely rare.

When corrosive media have direct access to metal substrate, corrosion electrochemical reactions will take place at the metal-coating interface in the presence of water and then delamination occurs. Hence, a good protective coating requires tight bonding between the coating and metal substrate to resist the penetration of water to the interface. The mechanical bond plays a dominant role in coating adhesion [27,28,29,30]. It is often used in engineering practices to enlarge the surface roughness of metals for increasing the contact area and improving adhesion between the coating and substrate metal. However, given that physical adsorption is the essence of the mechanical bond, this type of bond does not provide sufficient protection for long-term wet adhesion. Therefore, researchers have investigated various methods to enhance the wet adhesion of coatings. Chemical bonding is much stronger than physical adsorption, and the former can effectively impede lateral diffusion of water on the interface between the coating and metal [31,32]. Chemical bonding can maintain the wet adhesion and corrosion protection of coatings for a long time [10,33,34].

In this study, the swelling behaviors of two waterborne acrylic coatings were evaluated via SECM and the effects of adhesion mechanisms on the coating failure were discussed.

## 2. Results and Discussion

### 2.1. Characteristics of the Coating Structure

To analyze the characteristics of two resins after curing, the surface and cross-sectional shape of the two resins were observed by scanning electron microscopy (SEM) and atomic force microscopy (AFM). The contact angles of coatings were determined to analyze the hydrophilic character of the coatings. Infrared spectroscopy and secondary ion mass spectroscopy were combined to analyze the bonding between coating and metal.

**Figure 1:**
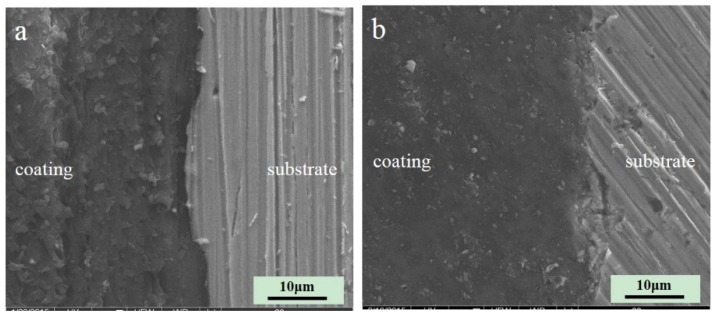
Section microstructure of the styrene-acrylic (**a**) and terpolymer (**b**) coatings.

Figure 1 shows the SEM micrograph of the cross-section of the two coatings. The styrene-acrylic coating is formed by styrene-acrylic latex, which has high barrier properties. The terpolymer coating, which contains acrylic acid (CH_2_=CH–COOH), vinyl chloride (CHCl=CH_2_), and 1,1-dichloroethylene (CH_2_=CCl_2_), has excellent adhesion performance. Both of them are single components and can be cured at normal atmospheric temperature. The interface of both coatings was intact without obvious defects, and the bonding between the coating and metal was close. Wrinkles caused by friction were observed on the cross-section of the styrene-acrylic coating. Under the same conditions, the cutting surface of the terpolymer coating was relatively smooth. Thus, the hardness of the terpolymer coating was softer than that of the styrene-acrylic coating [35].

**Figure 2:**
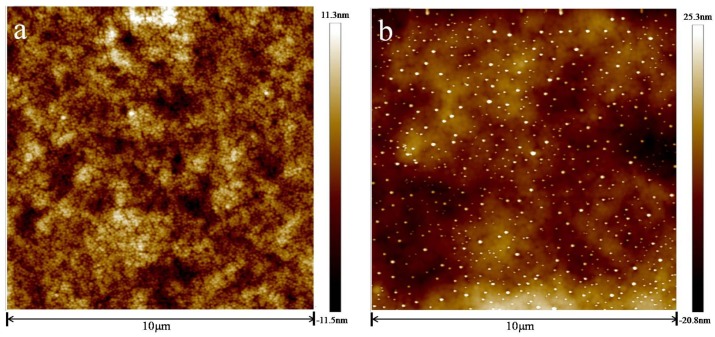
Initial microstructure of styrene-acrylic (**a**) and terpolymer (**b**) coatings.

Figure 2 shows the AFM images of the two coatings before immersion. Within the scanning range (10 μm × 10 μm), the morphology of both coatings was relatively flat and composed of a large number of small particles whose diameter was about 100 nm. This phenomenon was due to film-formation [36,37].

Upon comparing the morphology of the two coatings, the styrene-acrylic coating was smoother than the terpolymer coating. In addition, the terpolymer coating revealed a larger size and higher roughness than the styrene-acrylic coating.

**Figure 3:**
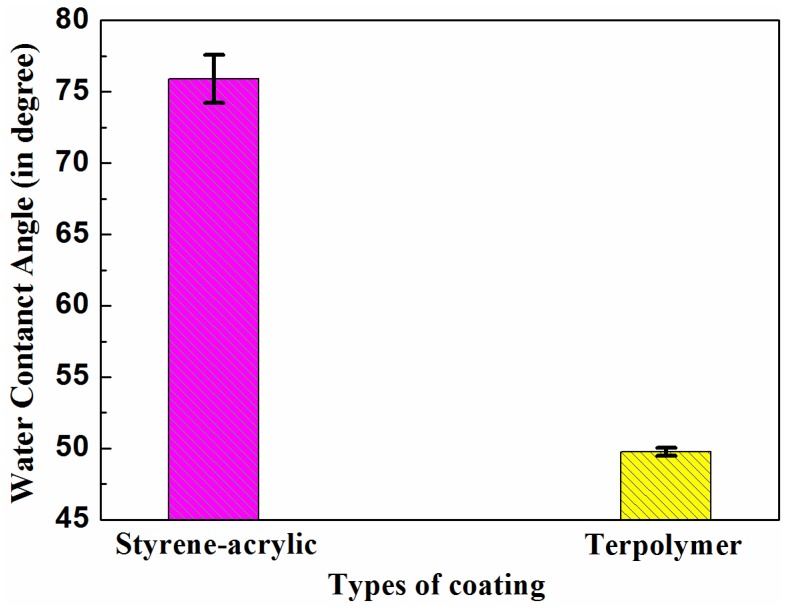
Contact angle of styrene-acrylic and terpolymer coating surfaces.

Figure 3 illustrates the contact angle test results of the two coating surfaces. The contact angles of the styrene-acrylic coating and terpolymer coating were 76° ± 1.68° and 50° ± 0.29°, respectively. The different contact angles of the two acrylic coatings indicated their difference in wettability. The styrene-acrylic coating demonstrated relatively high hydrophobicity. By contrast, the terpolymer coating was hydrophilic, which may be due to aggregation of the hydrophilic group surfactant on the coating surface [38].

**Figure 4:**
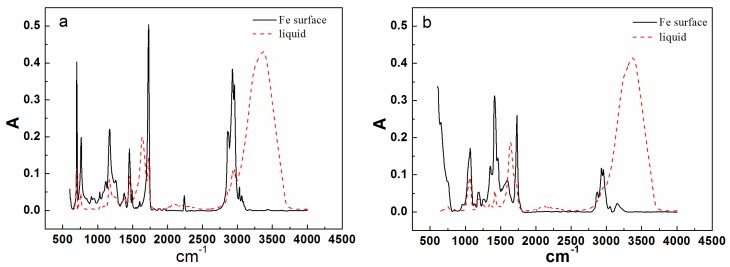
Infrared results of styrene-acrylic (**a**) and terpolymer (**b**) coatings.

Figure 4 shows the results of infrared analysis of the two resins and coatings in carbon steel substrate. When the coating was thin (less than 4 μm), infrared waves could traverse through it and reflect the composition of the coating/metal interface. For the terpolymer resin, a new peak formed in the vicinity of 1740 cm^–1^ after curing on the surface of carbon steel, which was due to the carbonyl produced by the reaction between the resin’s carboxyl groups and the matrix metal [39]. For the styrene-acrylic resin, no change in the original position was observed, except the peak intensity increased. To further verify the infrared results, the terpolymer coating on the carbon steel surface was analyzed by secondary ion mass spectrometry (SIMS).

**Figure 5:**
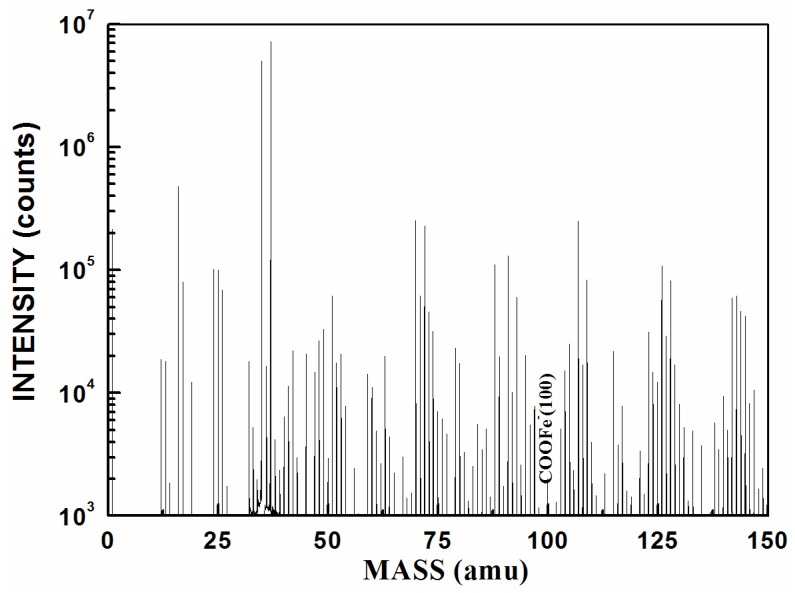
SIMS result of the interface between the terpolymer coating and metal.

In SIMS, ionized particles are ejected from the surface by the bombardment of a primary ion beam (Ar^+^, F^−^, O_2_^+^, O^−^, and Cs^+^) and then separated according to their masses. Both atoms and molecules can be ionized. Thus, details about the chemical state of atoms of the surface, such as bonding, are obtained. Figure 5 shows the SIMS result for the terpolymer coating on the carbon steel surface. The peak at 100 nm means that COOFe bonding existed on the coating/metal interface. This finding conformed to the results of infrared (IR) in which the surface between the terpolymer coating and the metal substrate exhibited a stable bond. However, no bonding occurred between the styrene-acrylic coating and the metal substrate.

### 2.2. Adhesion Force between the Metal and the Two Coatings

**Figure 6:**
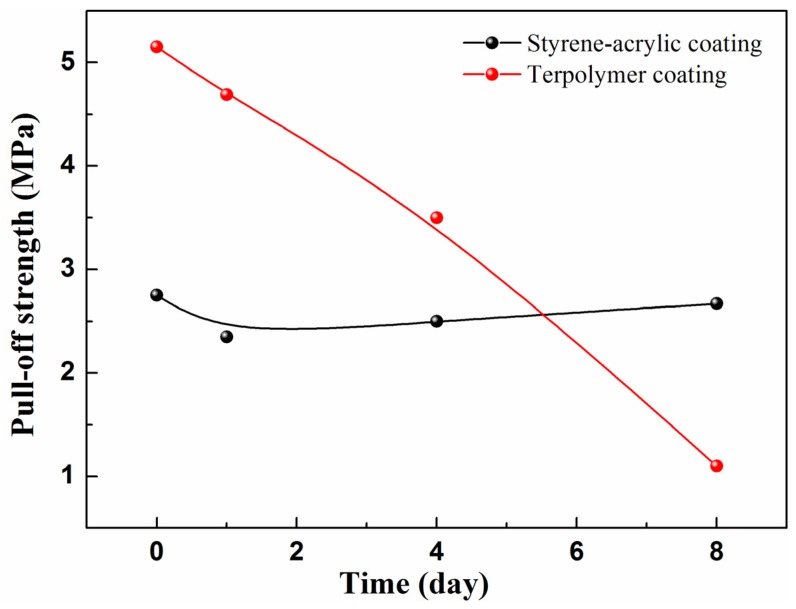
Wet adhesive force with the immersion time.

To evaluate the wet adhesive force of the coatings, a series of wet adhesion tests in the different immersion circles was performed. Figure 6 shows the wet adhesive force under different test cycles. The initial adhesive force of the styrene-acrylic coating is about 5 MPa which is almost twice that of the terpolymer coating. The terpolymer coating is too soft to withstand the pulling force [27,28]. However, with increasing immersion time, the adhesive force of the styrene-acrylic coating decreases rapidly, and the value is only 20% of the initial amount after eight days of immersion. By contrast, the terpolymer coating maintains its state. The terpolymer coating has a more reliable bonding in the coating/metal interface than the styrene-acrylic coating when the defect exists. This result can be attributed to the robust wet adhesive force which can effectively suppress the lateral diffusion of the corrosive ions to the coating/metal interface.

### 2.3. Microbubbling Process of the Coating Interface

SECM was conducted to test the micro bubbling process on the coating interface (all *X* and *Y* coordinates are in micrometers). According to Equation (1), a stable probe current was obtained in 3.5% NaCl solution for a probe potential of −0.7 V (SCE). When the probe approached the sample, the current presented the morphology of the surface of the sample. Therefore, the current could be used to characterize the morphology of the sample surface [24].

During analysis, the distance between the probe and sample was fixed at 40 µm and the test area was 0.25 mm^2^. To observe the changes in the sample in situ during immersion, the current signal of the sample surface was repeatedly tested within a certain time (4–24 h).

**Figure 7:**
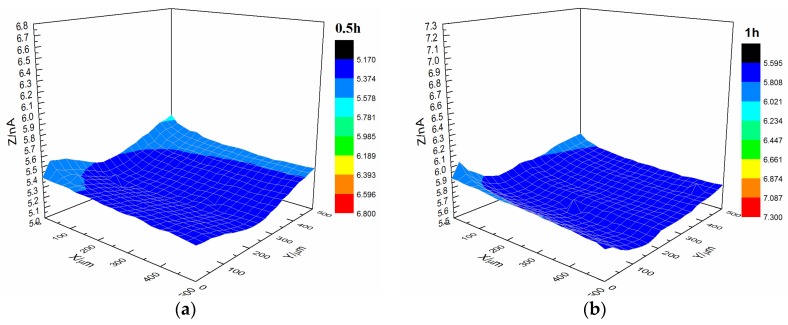

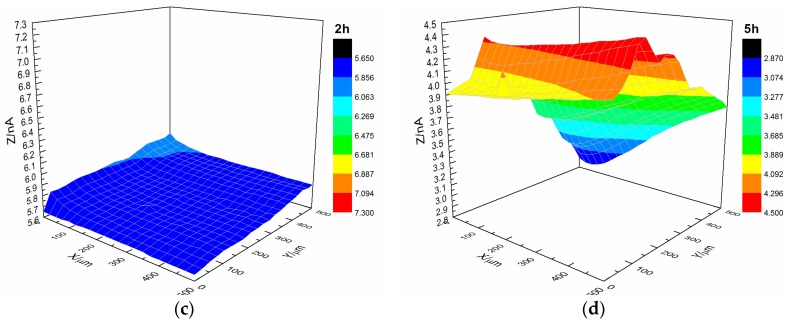
Current changes on the surface of the s-1 coating sample immersed in 3.5% NaCl solution. (**a**) 0.5 h, (**b**) 1 h, (**c**) 2 h and (**d**) 5 h.

Figure 7 shows the current changes on the surface of the s-1 coating sample immersed in 3.5% NaCl solution. The coating’s morphology was relatively flat with featureless points, and the range of variation of the current was 0.5 × 10^−9^ A after immersion for 0.5 h. By increasing the immersion time, the coating’s morphology changed. The central portion of the coating gradually changed from dark blue to red, and the current also increased to 1.7 × 10^−9^ A.

**Figure 8:**
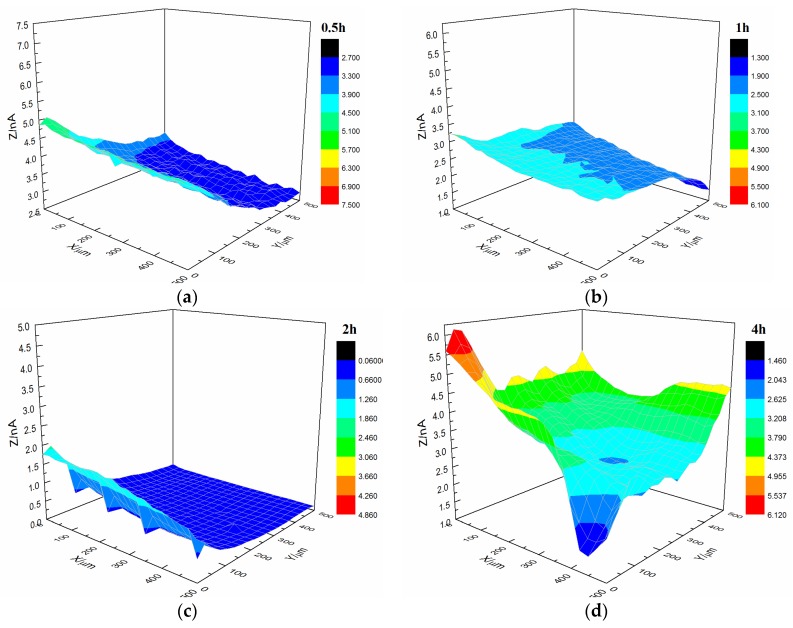
Current changes on the surface of the s-2 coating sample immersed in 3.5% NaCl solution. (**a**) 0.5 h, (**b**) 1 h, (**c**) 2 h and (**d**) 4 h.

Figure 8 shows the current changes on the surface of the s-2 coating sample immersed in a 3.5% NaCl solution. At the start of immersion, the coating’s morphology was relatively flat. By increasing the immersion time, the coating’s morphology changed. The SECM map of the coating transformed, and the current also increased to 4 × 10^−9^ A, which was higher than the current of s-1.

Upon comparing the surface changes in the s-1 and s-2 coating samples immersed in 3.5% NaCl solution, we found that the surface roughness of the substrate decreased, and the occurrence of microbubble formation declined. Thus, the bonding between the styrene-acrylic coating and metal weakened when the substrate surface roughness decreased. This result indicated that the bonding between the styrene-acrylic coating and metal was based on physical interactions.

**Figure 9:**
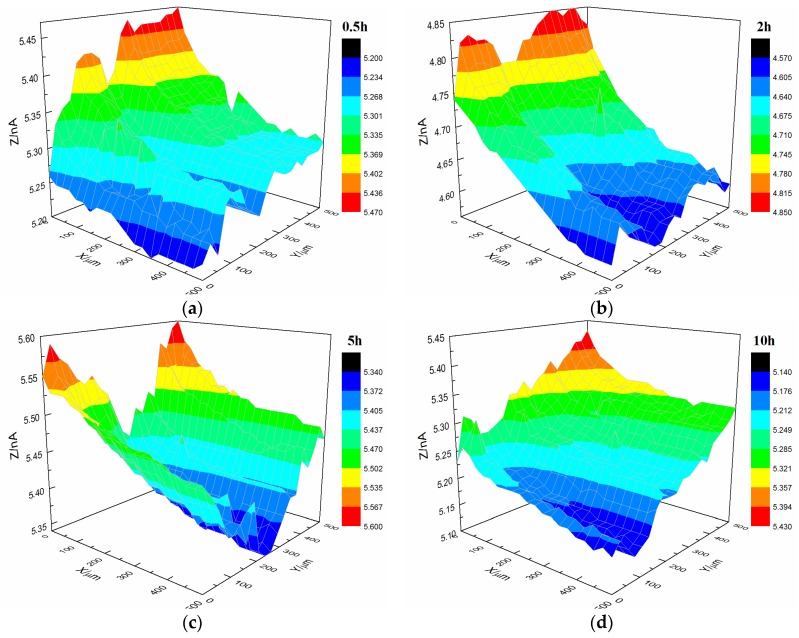
Current changes on the surface of t-1 coating sample immersed in 3.5% NaCl solution. (**a**) 0.5 h, (**b**) 2 h, (**c**) 5 h and (**d**) 10 h.

Figure 9 shows the current changes on the surface of the t-1 coating sample immersed in 3.5% NaCl solution. The morphology of the coating was relatively flat with featureless points, and the range of variation in the current value was 0.3 × 10^−9^ A after immersion for 0.5 h. The coating’s morphology did not change significantly after immersion for 10 h. The coating’s morphology was the same as the initial morphology, and the current remained at 0.3 × 10^−9^ A.

**Figure 10:**
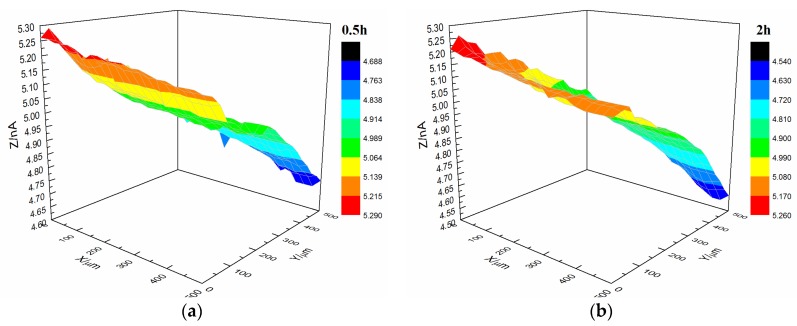

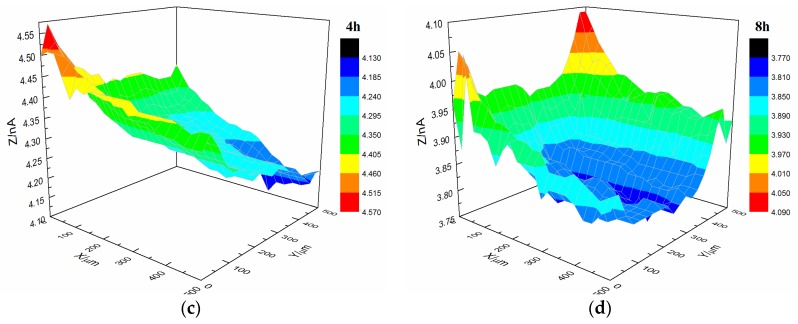
Current changes on the surface of the t-2 coating sample immersed in 3.5% NaCl solution. (**a**) 0.5 h, (**b**) 2 h, (**c**) 4 h and (**d**) 8 h.

Figure 10 shows the current changes on the surface of the t-2 coating sample immersed in 3.5% NaCl solution. Prior to immersion, the coating’s morphology was relatively flat with featureless points. The current was 0.5 × 10^−9^ A after immersion for 0.5 h. The coating’s morphology did not change significantly after immersion for 8 h. The coating’s morphology was the same as the initial morphology, and the current remained at 0.5 × 10^−9^ A.

Upon comparing the surface change of the t-1 and t-2 coating samples immersed in 3.5% NaCl solution, it can be found that chemical bonding played a major role between the terpolymer coating and metal substrate, and it was less affected by changes in surface roughness of the substrate. With respect to physical bonding, the samples were resistant to water and other media damage to the coating adhesion.

**Figure 11:**
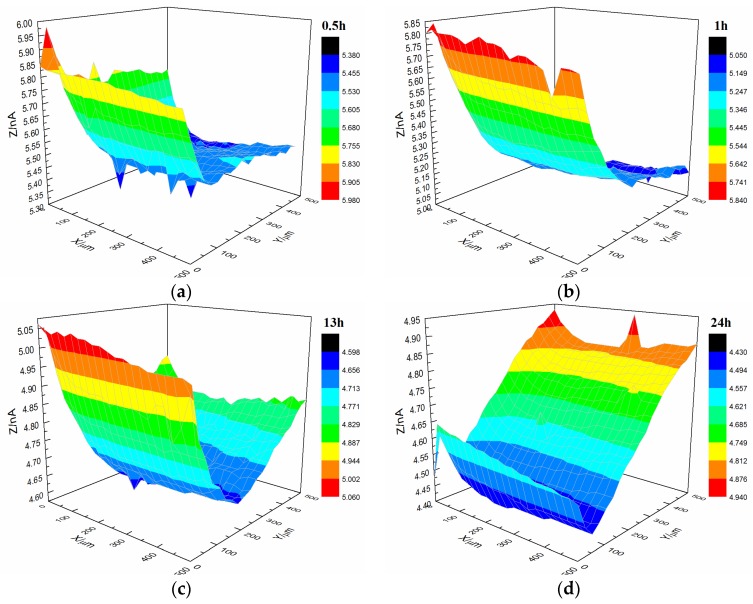
Current changes on the surface of the s-3 coating sample immersed in 3.5% NaCl solution. (**a**) 0.5 h, (**b**) 1 h, (**c**) 13 h and (**d**) 24 h.

Figure 11 illustrates the current changes on the surface of the s-3 coating sample immersed in 3.5% NaCl solution. After immersion for 13 h, the coating’s morphology was relatively flat with featureless points, and the current was 0.8 × 10^−9^ A. However, after immersion for 24 h, the coating’s morphology significantly changed, and the current of the upper and lower portions of the coating were altered. This finding indicated that microbubbles occurred in the coating.

**Figure 12:**
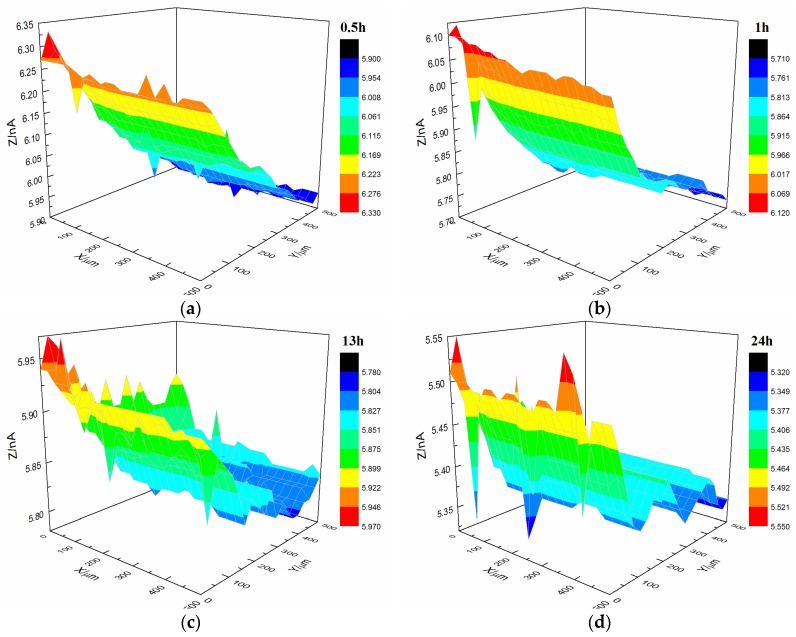
Current changes on the surface of the t-3 coating sample immersed in 3.5% NaCl solution. (**a**) 0.5 h, (**b**) 1 h, (**c**) 13 h and (**d**) 24 h.

Figure 12 shows the current changes on the surface of the t-3 coating sample immersed in 3.5% NaCl solution. After immersion for 24 h, the coating’s morphology was relatively flat with featureless points, and the current remained at 0.5 × 10^−9^ A.

### 2.4. Failure Mechanism of the Styrene-Acrylic and Terpolymer Coatings

Mechanical fitting is the most effective method for improving coating/metal adhesion. Before coating on the metal substrate, surface roughening treatment (such as sandblasting) on the metal substrate’s surface can increase the porosity of the surface so that paint can penetrate into the pores. The coating can rely on co-anchor, hooks, staples, and other forms of roots fixed on the metal substrate surface and firmly attached after curing, thereby enhancing the combined coating/metals. Such treatment result in physical bonding and improves the adhesion of the coating. The results of SECM analysis of styrene-acrylic showed that the coating on the 240# sandpaper-treated substrate exhibited microbubbles later than the coating on 2000# sandpaper-treated substrate.

Defects in the service life of a coating are inevitable. These defects will provide the channel for corrosive media, such as water, and expose the metal substrate to corrosive media. When the metal substrate begins to corrode, the anode and cathode area will be generated in these exposed areas randomly. Metal will dissolve at the anode zone and the pH will reduce. Oxygen will be reduced at the cathode zone and the pH will rise, which will promote coating delamination. The anode and cathode will separate because of aggravating corrosion, and delamination is extended [40]. Therefore after damage occurs, the activities at the interface between the coating and the steel surface are important for the mechanism of cathodic delamination. The interactions take place on top of a thin layer of ferrous oxide because steel surfaces prepared by abrasive blasting are oxidized instantaneously upon contact with the atmosphere [41]. Good barrier effects require robust adhesion between the coating and metal substrate. If good adhesion can be established, attack by water at the interface can be prevented [10,33]. As a consequence, tight bonding of a covalent or ionic character is necessary between the coating and metal. The results of infrared (Figure 4) and SIMS (Figure 5) analysis reveal COOFe bonding in the interface between the terpolymer coating and metal surface. The bonding energy of COOFe is stronger than hydrogen bonding of water and metal [39], so it can terminate the layers of water molecules formed on the surface of metal and improve the wetting adhesion of the coating.

**Figure 13:**
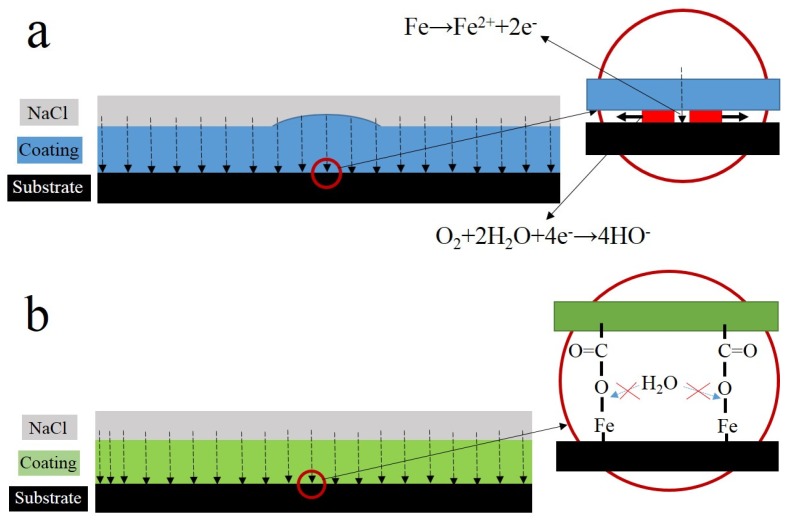
Schematic of the failure mechanism of the styrene-acrylic (**a**) and terpolymer (**b**) coating/metal interface.

Interface combination is mainly for physical adsorption (Figure 13a). Improving the metal surface roughness and increasing the contact area are effective ways to improve adhesion, but such methods for improving wet coating adhesion are limited. Although interface combination could directly increase the surface contact area between the coating and metal substrate, it failed to improve the water medium diffusion resistance in the coating/metal interface. The presented method only extended the lateral diffusion channel distance of the water medium and prolonged the time for microbubble formation, but the effect was limited (Figure 7 and Figure 8). Given that the mechanism of interface combination is chemical bonding (Figure 13b), metal surface roughness is not the main factor influencing the stability of interface bonding. COOFe bonding can effectively prevent the lateral diffusion of the water medium in the metal/coating interface, so it can prolong the time of microbubble formation [42,43,44,45]. Under different roughness values, the terpolymer coating showed good wet adhesion (Figure 9 and Figure 10). Compared with the styrene-acrylic coating, the terpolymer coating exhibited better wet adhesion in different surfaces. The coating/metal interface of the terpolymer coating was mainly combined with chemical bonds and minimally affected by the roughness of the metal surface because the bonding energy of COOFe is stronger than the hydrogen bond between water and metal. The metal substrate’s surface roughness and interface bonding ways are critical factors that affect the stability of the combination of the coating/metal interface, but the influence of the substrate surface roughness on wet adhesion is limited, and bonding is crucial for wet adhesion.

Upon comparing the surface changes in the s-1 and s-3 coating samples immersed in 3.5% NaCl solution (Figure 7 and Figure 11), water permeation was delayed with increasing coating thickness. Thus, the change in the styrene-acrylic coating occurred later. However, when corrosive media passed through the coating and entered the coating/metal interface, the coating/metal interface failed to resist microbubble formation.

AFM observations revealed that the microstructure of the styrene-acrylic coating was denser than that of the terpolymer coating (Figure 1). Simultaneously, the contact angle test showed that the terpolymer coating exhibited certain hydrophobicity, which was disadvantageous for the penetration resistance of the coating. These findings indirectly illustrate that the penetration resistance of the styrene-acrylic coating was stronger than that of the terpolymer coating. When the surface changes in s-3 and t-3 were compared (Figure 11 and Figure 12), we noted that the increased thickness of the coating extended the longitudinal diffusion time of the water medium and delayed the time for microbubble formation. However, the interface stability of the terpolymer coating was still superior to that of the styrene-acrylic coating. Therefore, the permeability resistance of the coating did not affect the stability of the coating/metal interface. The bonding of the coating/metal interface is a key factor affecting the stability of the coating/metal interface.

Effective chemical bonds in the coating/metal interface are essential to resist coating damage and improve the coating’s service life.

## 3. Materials and Methods

### 3.1. Materials

In this paper, we used two water-based coatings, namely, styrene-acrylic coating and terpolymer coating consisting of acrylic acid (CH_2_=CH–COOH), vinyl chloride (CHCl=CH_2_), and 1,1-dichloroethylene (CH_2_=CCl_2_).

### 3.2. Sample Preparation

The samples for SECM were explained in Table 1. Q235 carbon steel, which was used as a metallic substrate, was polished by 240# sandpaper to remove the surface oxide layer. Some samples were polished successively by 240#, 400#, and 2000# to achieve different surface roughness. After polishing, the metal surface was carefully washed in ethanol and acetone and then dried prior to the coating process. The water-based acrylic acid coating was painted on the metallic surface with a brush, and the metal was cured at room temperature for 15 days. The thicknesses of the dry film were controlled at 10 and 20 μm. The measuring device (QNix4500, Automation Dr. Nix GmbH & Co.KG, Cologne, Germany, 0–50 μm ≤ ±1 μm) was used to control the thicknesses of the dry film, and both of the coatings have good liquidity and leveling.

### 3.3. Characterization of the Coating Properties

The coating properties were characterized by observing the morphology and measuring the contact angle, and analyzing the chemical changes via FTIR (PerkinElmer, Waltham, MA, USA). Moreover, SIMS (ION-TOF, Munster, Germany) was conducted to analyze the chemical composition of the coating/metal interface. SECM (Bio-Logic, Seyssinet-Pariset, France) was performed to measure the surface current. The current distribution on the surface of the coating was measured with different times and the change in the current distribution was obtained to characterize the development of micro-bubbles.

#### 3.3.1. Morphology

The section and surface morphologies of the coatings were observed via scanning electron microscopy (SEM, QUANTA 250, FEI, Hillsboro, OR, USA) and atomic force microscopy (AFM MultiMode^TM^ Nanoscope V, Bruker, Madison, WI, USA).

#### 3.3.2. Contact Angle

The contact angles of the coatings were measured using a DataPhysics contact angle measuring system (OCA 20, DataPhysics, Stuttgart, Germany). The droplet volume was 5 μL.

#### 3.3.3. FTIR Analysis

Chemical changes in coatings were monitored by Attenuated Total Reflectance (ATR) FTIR analysis using a PerkinElmer Frontier spectrometer in the range of 4000–650 cm^−1^ with 16 scans and a resolution of 4 cm^−1^. Changes between the resin and coating were tested to study the bond between the coating and metal. The samples were made by the spreader (OSP-04, OSP, Aichi Prefecture, Japan). The thicknesses of the dry film were less than 2 μm.

#### 3.3.4. SIMS

An ION-TOF GmbH TOF-SIMS 5 TOF ion mass spectrometry system (ION-TOF) was used to analyze the chemical composition of the coating/metal interface.

#### 3.3.5. Adhesion Testing

The sample size was 50 mm × 150 mm and the thickness of the coating was 85 ± 3 μm. A 20 mm width defect in the center of the sample was produced by an art knife, and the metallic substrates were exposed to air. In this study, the scratch depths of all metallic substrate were the same. The samples were immersed in 3.5 wt% NaCl solution (pH 7) at 30 °C with different immersion times. Subsequently, the samples were taken out and placed in 50% humidity and 25 °C for 2 h. A pillar was then bonded to the sample surface, and the distance between the center of the pillar and the defect was 25 mm. Prior to the test, the sample was placed in 50% humidity and 25 °C for 24 h to ensure a tight bond between the pillar and the sample. The wet adhesion test was performed by the PosiTest AT Pull-Off Adhesion Tester (DeFelsko, New York, NY, USA). The diameter of the pillar was 20 mm.

#### 3.3.6. SECM Measurements

SECM measurements were carried out over a large surface area (e.g., 0.25 mm^2^) as a function of immersion time. SECM scans were acquired by rastering over the sample surface in steps of 50 µm in the *Z*-direction, 500 µm in the *Y*-direction, and 500 µm in the *X*-direction. All of the experiments were conducted at room temperature (25 °C) in a naturally-aerated cell consisting of 3.5% NaCl solution. The oxygen dissolved in the electrolytic phase was employed as redox mediator for SECM imaging:

O_2_ + 2H_2_*O* + 4e^−^ ↔ 4OH^−^ (1)

## 4. Conclusions

Characteristics of the physical structure of coatings were studied by SEM and AFM. FTIR and SIMS were conducted to characterize the bonding between the terpolymer coating and metal substrate. SECM was used as a tool for the accelerated investigation of coating samples by measuring topographic changes in the exposed surface as a function of elapsed time.

The effects of thickness, surface roughness, and chemical bonding on the rate of cathodic delamination were investigated to gain further insight into the detailed mechanism of cathodic delamination and help optimize the coating formulation against cathodic delamination. Increasing the surface roughness and thickness could help adhesion, but the effect was limited. COOFe bonding is an efficient technique to improve wetting adhesion and prevent attack by water at the interface. It can also promote anticorrosion.

## Figures and Tables

**Table 1:** Designation, film thickness, and surface roughness of the tested coating systems.

Number	Coating Species	Coating Thickness (μm)	Surface Roughness
**s-1**	styrene-acrylic	10	240# (Ra0.673)
**s-2**	styrene-acrylic	10	2000# (Ra0.088)
**s-3**	styrene-acrylic	20	240# (Ra0.673)
**t-1**	terpolymer	10	240# (Ra0.673)
**t-2**	terpolymer	10	2000# (Ra0.088)
**t-3**	terpolymer	20	240# (Ra0.673)
